# *Carex meyeriana* Kunth Extract Is a Novel Natural Drug against *Candida albicans*

**DOI:** 10.3390/ijms25137288

**Published:** 2024-07-02

**Authors:** Panpan Du, Bingyan Liu, Xueting Wang, Zhong Zheng, Shu Liu, Songlei Guan, Zong Hou

**Affiliations:** 1 School of Life Sciences, Jilin Agricultural University, Changchun 130118, China; 18843139556@163.com (P.D.); 15643893056@163.com (B.L.); w43508081@163.com (X.W.); 2 Changchun Institute of Applied Chemistry, Chinese Academy of Sciences, Changchun 130022, China; zhengzh@ciac.ac.cn (Z.Z.); mslab20@ciac.ac.cn (S.L.); houzong@ciac.ac.cn (Z.H.)

**Keywords:** *Carex meyeriana* Kunth, *Candida albicans*, vaginitis, metabolomics

## Abstract

As a widely distributed plant in Northeast China, *Carex meyeriana* Kunth (CMK) is generally considered to have antibacterial properties; however, there is a lack of scientific evidence for this. Therefore, we investigated the chemical composition of CMK extract and its effect against *C. albicans.* A total of 105 compounds were identified in the alcohol extracts of CMK by UPLC-Q-TOF-MS. Most were flavonoids, with *Luteolin* being the most represented. Among them, 19 compounds are found in the *C. albicans* lysates. After treatment with CMK ethanol extract, a significant reduction in the number of *C. albicans* colonies was observed in a vaginal douche solution from day 5 (*p* < 0.05). Furthermore, the CMK extract can reduce the number of *C. albicans* spores. The levels of IL-4, IL-6, IL-10, IL-1β, and TNF-α in vaginal tissues all exhibited a significant decrease (*p* < 0.05) compared to those in the model group as determined by ELISA. The results of HE staining showed that CMK extract can eliminate vaginal mucosa inflammation. CMK adjusts the vaginal mucosa cells by targeting twenty-six different metabolites and five specific metabolic pathways in order to effectively eliminate inflammation. Simultaneously, the CMK regulates twenty-three types of metabolites and six metabolic pathways against *C. albicans* infection. So, CMK strongly inhibits the growth of *C. albicans* and significantly reduces vaginal inflammation, making it a promising candidate for treating *C. albicans* infection.

## 1. Introduction

*Candida albicans* (*C. albicans*) is a normal part of the human body’s biome, and it is primarily located on the skin’s surface, in the mouth, and in the vagina. When the body’s immune system is compromised, *C. albicans* can become a pathogen, leading to infectious diseases [1]. *C. albicans* vaginitis accounts for 80–90% of fungal vaginitis infections in China [2]. Due to the overuse of antifungal drugs in China, there is a serious issue of fungal drug resistance [3,4,5]. Therefore, there is an urgent need to discover new antifungal drugs.

*Carex meyeriana* Kunth (CMK) is a species of sedge grass that is distributed in the regions of Amur, Buryat, Central China, Inner Mongolia, Irkutsk, Japan, Khabarovsk, North Korea, Manchuria, Mongolia, Primoye (Primorsky Krai), Western Siberia, and Yakutsk. The plant is triangular, hard, brown, and shiny. The leaves are thin and tough. Male flowers are terminal, cylindrical, and have lateral spikelets, and female flowers are spherical or ovoid; the fruit sac is equal to or slightly longer than the scales, oval or elliptic, and the nutlets are tightly wrapped in the fruit sac, obovate elliptic, and brown. The flowering and fruiting period is from June to July. The plant grows in warm, sunny and wet environments. It grows in wet meadow environments with perennial ponding, and its growing soil is mainly swamp soil. The mode of reproduction is seed reproduction.

The entire plant, CMK, is one of northeast China’s three treasures [6]. CMK is a natural plant with abundant resources and a high medicinal development value; it has been found to have effects such as eliminating mites, activating collateral circulation, and improving immunity. In recent years, its antibacterial and antifungal effects have garnered significant attention [7]. However, there is a lack of consistent conclusions regarding the antibacterial and antifungal mechanisms of CMK. The lack of in-depth studies of the mechanism may be attributed to an incomplete understanding of the chemical composition. The current studies mainly believe that flavonoids, alkaloids, fatty acids, and volatile oils are the most abundant components of CMK, and luteolin is the main component with pharmacological activity [7,8].

Therefore, in this study, the mechanism of CMK in preventing *C. albicans* vaginitis infections was analyzed through detailed chemical composition analysis, efficacy testing of fungi inhibition, metabolomics of rat vaginal mucosal tissue, microbial metabolomics and other methods. The findings of this study suggest that CMK may serve as a promising candidate to be an antifungal agent against *C. albicans*.

## 2. Results

### 2.1. Chemical Composition of Ethanol Extract from Carex meyeriana Kunth

**Figure 1:**
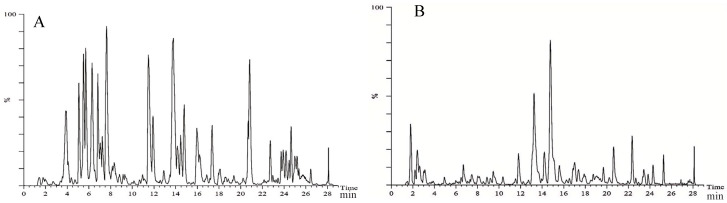
Total ion flow diagrams of the CMK ethanol extract. (**A**) Positive ion mode; (**B**) Negative ion mode.

**Figure 2:**
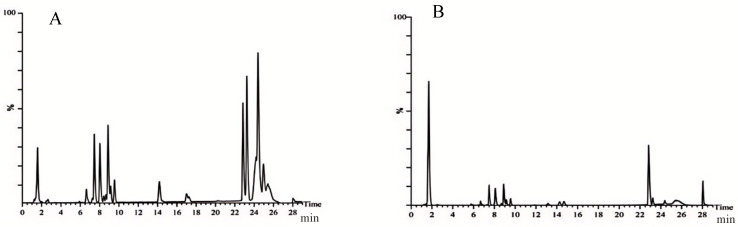
Total ion flow diagram of the *C. albicans* lysis solution. (**A**) Positive ion mode; (**B**) Negative ion mode.

CMK ethanol extract was used to identify a total of 105 compounds, including 33 flavonoids, 24 organic acid compounds, 14 phenylpropanoid compounds, 14 terpenoid compounds, and another 20 compounds. These findings will serve as the groundwork for future research on the therapeutic properties of the ethanolic extract of CMK. More importantly, a total of 19 original chemical components were found in *C. albicans* lyastes, primarily flavonoids. These components include neotigogenin acetate, luteolin, echinothiophene, trifolin, orientin, rutin, tracheloside, β-Hydroxyisovalerylshikonin, tiliroside, plantamajoside, sinensetin, isodemethylwedelolactone, tricin, isosilybin, wedelolactone, 9,16-Dioxyhydroxy-10,12,14 triene18 carbonic acid, and 6-gingerol Methyl lucidenate P. These components are most likely the potent chemicals in CMK that act against *C. albicans*. Please refer to Figure 1 and Figure 2 and Table 1, as well as Tables S8 and S9 in the Supplementary Materials, for more details.

### 2.2. The Inhibitory Effect of Carex meyeriana Kunth against C. albicans

**Figure 3:**
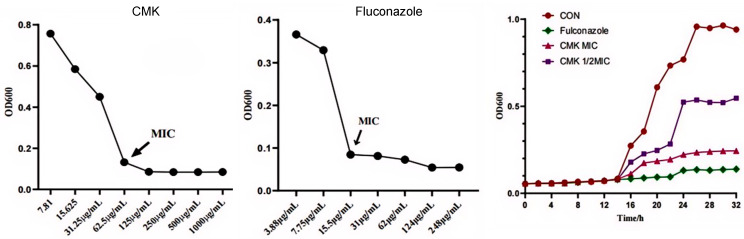
The growth inhibition effect of CMK on *C. albicans*. MIC of CMK is 62.5 µg/mL. MIC of fluconazole is 15.5 µg/mL.

The MIC of CMK is only 62.5 µg/mL (Figure 3). After 14 h, the inhibition rates significantly increase. Specifically, the inhibition percentage was 89.62% at the 24 h mark, and the highest percentage was 91.14% at the 26 h mark. This indicates that CMK strongly inhibited the growth and proliferation of *C. albicans.* Although MIC value is higher than that of fluconazole, as a natural plant component, it has a strong antifungal effect against *C. albicans*.

**Figure 4:**
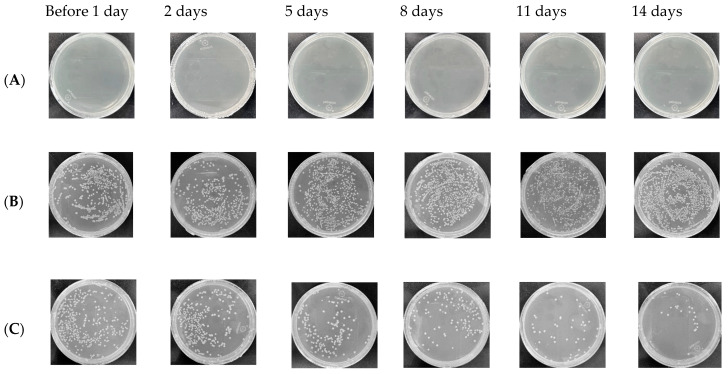

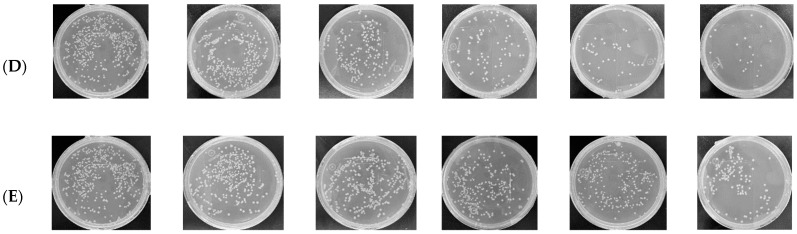
The *C. albicans* colonies. Rat vaginal lavage solution coated on LB solid medium. (**A**) Control group. (**B**) Model group; (**C**) FCZ group; (**D**) high-dose group; (**E**) low-dose group. After 5 days, the number of colonies of the FCZ group and high dose groups significantly decreased.

**Figure 5:**
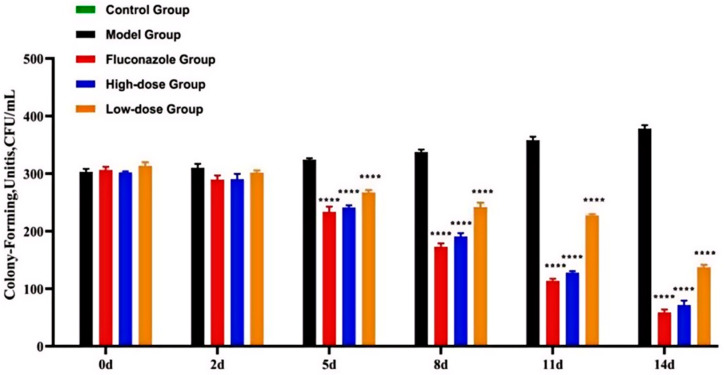
The number of *Candida albicans* colonies in vaginal flushing fluid. Compared to the model group, the difference is statistically significant (**** *p* < 0.0001).

Figure 4 and Figure 5 illustrate colonies derived from rat vaginal lavage fluid. After 5 days, the number of vaginal colonies in the FCZ group, the high-dose group, and the low-dose group significantly decreased compared to those in the model group (*p* < 0.0001). No colonies formed in the control group.

**Figure 6:**
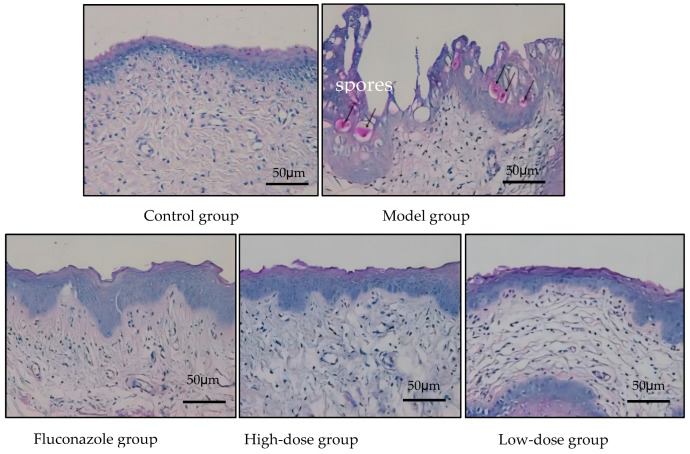
The pathological sections of vaginal tissue of the rats are stain with PAS (×200). In model group, black arrows indicate spores present in the vaginal tissues. However, there is no significant infiltration of *C. albicans* spores observe in the vaginal tissues of rats treat with fluconazole or high-dose CMK. Only two rats in the low-dose group show a small amount of spore infiltration in their vaginal tissues, as indicate by PAS staining.

The epithelial cell layer in the model group exhibited thickening, with localized shedding or the absence of epithelial cells. The epithelial layer test PAS+, and circular small granular spores are dispersed within the epithelial layer. However, no PAS+ spores were detected in either the FCZ group or the high-dose group, although two rats in the low-dose group displayed spores. Edematous changes in subepithelial tissues were observed in the FCZ group; however, there were no significant edematous changes in the subepithelial stroma in the high-dose group. Tissue inflammation was significantly reduced in both the low-dose and high-dose groups compare to the model group (Figure 6).

### 2.3. The Anti-Inflammatory of Carex meyeriana Kunth

#### 2.3.1. The Levels of Inflammatory Mediators Are Decrease

**Figure 7:**
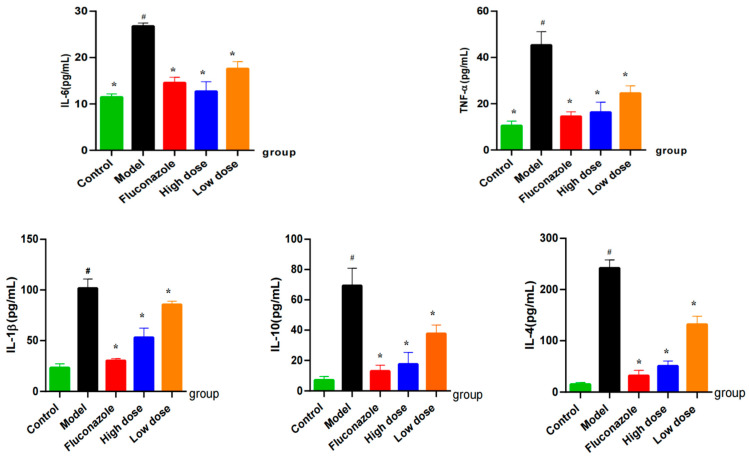
Levels of IL-4, IL-6, IL-10, IL-1β, and TNF-α in vaginal tissues of vaginitis model rats. Comparison with the control group, # *p* < 0.05; comparison with the model group, * *p* < 0.05.

The levels of IL-4, IL-6, IL-10, IL-1β, and TNF-α in the vaginal tissue of the model group were significantly higher than those in the control group (*p* < 0.05). These results indicate that these inflammatory mediators are associated with *C. albicans* infection. However, after treatment with CMK (regardless a high or low dose) or FCZ, the levels of IL-4, IL-6, IL-10, IL-1β, and TNF-α decreased significantly (*p* < 0.05). Furthermore, the levels of TNF-α, IL-6, IL-4, and IL-10 in the vaginal tissues were found to be similar between the high-dose and FCZ groups (Figure 7), providing further evidence that the anti-inflammatory effect of CMK extract closely resembles that of FCZ, which is commonly use in clinical practice.

#### 2.3.2. Eliminating Inflammation of Vaginal Mucosa Tissue

We observed that the vaginal mucosa tissue of the healthy control group of rats exhibited even HE staining, with smooth mucosa, distinct tissue layers, neatly arranged epithelial cells, and no infiltration of inflammatory cells. In contrast, the vaginal mucosa epithelium of the model group showed excessive proliferation and abnormal keratinization of epithelial cells, as shown by the HE staining. Additionally, significant mucosa edema was present in the subepithelial stroma, accompanied by the infiltration of inflammatory cells such as neutrophils and lymphocytes. Furthermore, a large amount of capillary dilation and congestion was evident in the vaginal tissue.

**Figure 8:**
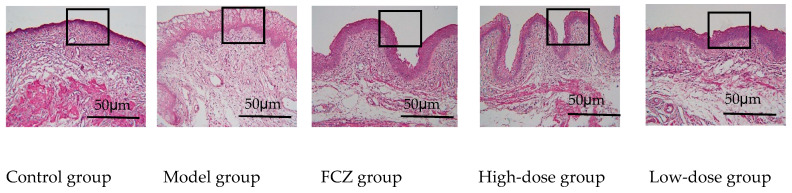
Pathological section of vaginal tissue of rats. Biopsies were stained with HE, ×200. The control group’s mucosa is made up of smooth, distinct tissue layers, without inflammatory cell infiltration, and the cells are arranged neatly. The model group’s vaginal tissues show severe edema, cell deformation, telangiectasia, and inflammatory cell infiltration in the submucosa. The vaginal mucosal tissue edema of rats in the FCZ group, high-dose group, and low-dose group was significantly reduced, inflammatory cell infiltration was significantly reduced, and the vascular dilation and congestion disappeared.

However, in the treatment groups, the vaginal mucosa tissues of rats displayed signs of repair or healing. The numbers of infiltrate inflammatory cells in the subepithelial interstitium were reduced and mucosa edema in this area was effectively ameliorated. The vaginal mucosal tissue layer was restored (Figure 8). Following the elimination of vaginal inflammation, the weight of the rats gradually recovered (Supplementary Materials Figure S1).

#### 2.3.3. Metabolites and Metabolic Pathways for Anti-Inflammatory in Vaginal Tissue

(1) Prediction of potential targets in non-targeted metabolomics of vaginal tissue

Significant differences were observed in the metabolic profiles between groups (Supplementary Materials Figure S2). To identify potential targets, OPLS-DA in positive and negative ion scanning modes was established and S-plots were generated (Supplementary Materials Figure S3). A total of 26 endogenous metabolites were identified through the multivariate analysis. Following vaginal lavage, six of the metabolites illustrated a significant upregulation, while fourteen metabolites exhibited a significant downregulation (Table 2).

(2) Non-targeted metabolic pathways in vaginal tissue

**Figure 9:**
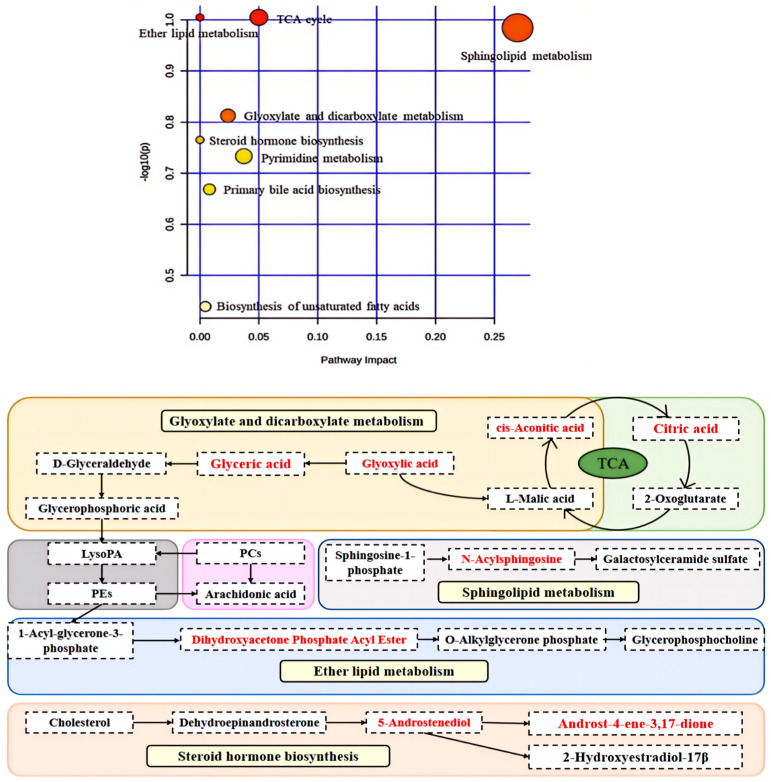
The metabolic networks of major metabolites in vaginal tissue. In the bubble chart, the redder and the larger the bubble size, the more significant the changes in potential biomarkers of metabolic pathways such as ether lipids metabolism, TCA cycle, sphingolipid metabolism, glyoxylate and dicarboxylate metabolism, and steroid hormone biosynthesis. Based on the bubble map, the metabolic network of the aforementioned five metabolic pathways has been delineated. Glyoxylate and dicarboxylate metabolism of Glyoxylic acid to L-Malic acid and Glyceric acid, respectively; L-Malic acid metabolism of cis-Aconitic acid, and the further metabolism of cis-Aconitic acid to citric acid. Citric acid metabolizes 2-Oxoglutarate, and 2-Oxoglutarate can be metabolized to L-Malic acid through the TCA cycle to provide energy for the body. Glyceric acid is metabolized to Glyceric acid, Glyceric acid is further metabolized to D-Glyceraldehyde, D-Glyceraldehyde is metabolized to Glycerophosphoric acid, and Glycerophosphoric acid is metabolized to LysoPA, which is further metabolized to PEs, which are metabolized to Arachidonic acid and 1-Acyl-glycerone-3-phosphate, respectively. PCs can be metabolized to Arachidonic acid and LysoPA, respectively. 1-Acyl-glycerone-3-phosphate in Ether lipid metabolism can be metabolized to Dihydroxyacetone Phosphate Acyl Ester. Dihydroxyacetone Phosphate Acyl Ester is further metabolized to O-Alkylglycerone phosphate. Finally, O-Alkylglycerone phosphate was metabolized to Glycerophosphocholine. Sphingolipid is metabolized to Sphingosine 1-phosphate which is further metabolized to N-Acylsphingosine, and N-Acylsphingosine metabolizes to Galactosylceramide sulfate, thus maintaining a normal physiological state. Cholesterol is metabolized by the steroid hormone biosynthesis to Dehydroepinandrosterone, and Dehydroepinandrosterone can be further metabolized to 5-Androstenediol; 5-Androstenediol is finally metabolized to Androst-4-ene-3,17-dione and 2-Hydroxyestradiol-17β, which resist the invasion of pathogenic microorganisms. These metabolic pathways directly affect the energy metabolism process of vaginal mucosa or mucosal immune cells.

There are twenty-six metabolites that are potentially targeted by CMK in vaginal tissue, which disrupts eight metabolic pathways and thus inhibits inflammation. The metabolic network is illustrated in Figure 9, indicating that these metabolites mainly belong to several metabolic pathways such as glyoxylate and dicarboxylic acid metabolism, the citric acid cycle (TCA cycle), ether lipid metabolism, sphingolipid metabolism, or steroid hormone biosynthesis. CMK may interfere with 26 metabolites, thereby suppressing the mucosal inflammation.

### 2.4. Metabolites and Metabolic Pathway of Carex meyeriana Kunth against C. albicans

#### 2.4.1. Prediction of Potential Targets in Non-Target Metabolomics of *C. albicans*

Significant differences were observed in the metabolic profiles between groups (Supplementary Materials Figure S4). OPLS-DA was established in the positive and negative ion scanning modes and S-plots were generated (Supplementary Materials Figure S5). A total of 23 endogenous metabolites were identified through multivariate analysis. Following drug treatment, nine metabolites showed a significant upregulation, while fourteen metabolites exhibited a significant downregulation (Table 3).

#### 2.4.2. Non-Target Metabolic Pathways in *C. albicans*

**Figure 10:**
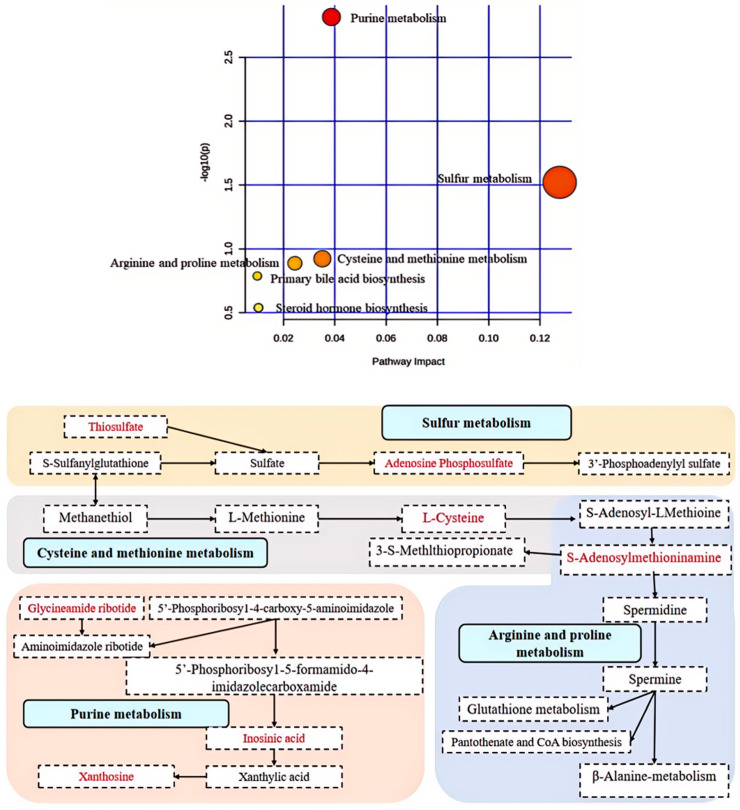
The metabolic networks of major metabolites of *C. albicans*. In the bubble chart, the redder the bubble and the larger the bubble size, the more significant changes in potential biomarkers of metabolic pathways such as purine metabolism, sulfur metabolism, cysteine and methionine metabolism, arginine and proline metabolism. Based on the bubble map, the metabolic network of the aforementioned five metabolic pathways has been delineated. In sulfur metabolism, sulfate is metabolized by thiosulfate and S-sulfanylglutathione. Sulfate is further metabolized to adenosine phosphosulfate, which eventually converts to 3′-phosphoadenylyl sulfate. Meanwhile, S-sulfanylglutathione is metabolized to methanethiol, which in turn is further metabolized to L-methionine. L-methionine then undergoes further metabolism to L-cysteine, followed by conversion to S-adenosyl-L-methioine. This compound is subsequently metabolized to S-adenosylmethioninamine, finally leading to the production of 3-S-methylthiopropionate and spermidine. Spermidine itself goes through a metabolic process that results in its conversion into spermine. Eventually, this leads to involvement in glutathione metabolism, pantothenate and CoA biosynthesis, as well as β-alanine metabolism. These processes directly regulate the growth and reproduction of *C. albicans.* In purine metabolism, aminoimidazole ribotide derives from glycineamide ribotide and 5′-phosphoribosy1-4-carboxy-5-aminoimidazole. The latter can be converted into aminoimidazole ribotide or 5′-phosphoribosy1-5-formamido-4-imidazolecarboxamide. The latter compound then undergoes further metabolism into inosinic acid before being converted into xanthylic acid as part of xanthylic acid metabolism for xanthosine. The above metabolic pathways directly interfered with the nucleic acid and protein synthesis of *C. albicans*.

Twenty-three metabolites are potentially targeted by CMK in *C. albicans*, and it was found that six metabolic pathways were disrupted and regulated. A metabolic network was constructed, as depicted in Figure 10, indicating that the potential metabolites mainly belong to several metabolic pathways such as purine metabolism, thiol metabolism, cysteine and methionine metabolism, arginine and proline metabolism, etc. CMK interferes with the purine and amino acid metabolism of *C. albicans*, affects nucleic acid and protein synthesis, and leads to its death.

## 3. Discussion

*C. albicans* vaginitis is a predominant female genital tract infection closely associated with various gynecological complications in women of reproductive age, accounting for 80% to 90% of all cases of vaginitis. Symptoms include increased vaginal discharge, swelling of the external genitalia, itching, and a burning sensation, significantly impacting women’s health and quality of life. At present, there are many kinds of drugs used for the treatment of *Candida albicans* infectious vaginitis, mainly including itraconazole, fluconazole, nystatin, clotrimazole, or miconazole, etc. [9,10,11]. However, due to the escalating issues of drug resistance and abuse, there is an urgent need to explore new antifungal medications [12,13]. Thus, extensive experiments have been conducted by scientists in this regard. Zida et al. identified 142 natural products with potential activity against *C. albicans*; most were derived from plants native to Asia and the United States. Additionally, 16 of these natural products had their antifungal activity confirmed through in vivo experimentation [14]. Gao et al. also discovered that quercetin, a dietary flavonoid, exhibited promising antifungal activity against *C. albicans* by inhibiting fungal adherence and biofilm formation [15]. These findings suggest that numerous natural products have promising clinical value in the treatment of vaginal candidiasis.

As a widely available plant with abundant resources and affordable prices, CMK is recognized as one of the “three treasures” in northeast China, alongside ginseng and deer antlers. It possesses a high application value and has been traditionally utilized to make insoles, pillows, mattresses, and other daily necessities for centuries. In ancient times, CMK was commonly used for insoles to provide warmth and prevent various fungal foot diseases when placed inside shoes [16]. However, due to limited research reports, the development of CMK products has been hindered, resulting in an underutilization of its medicinal benefits.

Su Ting utilized CMK extracts in the treatment of oral diseases caused by *C. albicans*, and the oral cavity recovered well. Only 94 chemical compounds were identified in Su Ting’s study. However, we identified 105 chemical compounds, due to the use of higher resolution scanning instruments and identification software, and most of which were different from those of Su Ting et al. [7]. The method used in this research has a higher sensitivity, resulting in more comprehensive and credible results. Therefore, these data provide a more comprehensive database of compounds for the medicinal development of CMK. Out of the 105 compounds identified, each ingredient exhibited pharmacological activity, indicating that CMK has a wide range of medicinal applications. In addition, the 19 compounds in the lysates may be the natural ingredients against *C. albicans*.

We observed that CMK inhibited the growth and proliferation of *C. albicans* by interfering with twenty-three metabolites and six types of metabolic pathways, including purine metabolism, sulfur metabolism, cysteine and methionine metabolism, and arginine and proline metabolism. These pathways are closely associated with nucleic acid and protein synthesis, directly interfering with the growth and reproduction of *C. albicans*. It is possible that CMK inhibits the growth of *C. albicans* by interfering with these key metabolic pathways. All these metabolites are potential regulatory targets for CMK inhibition of *C. albicans*.

We also performed a non-targeted metabolomics analysis on rat vaginal tissue and identified 26 metabolites. We identified five types of metabolic pathways closely related to vaginitis: glyoxylate and dicarboxylic acid metabolism; the citric acid cycle (TCA cycle); ether lipid metabolism; sphingolipid metabolism; and steroid hormone biosynthesis. Both sphingolipid metabolism and ether lipid metabolism are crucial for lipid metabolism, which is closely related to inflammation. Lipids can exert anti-inflammatory actions by interfering with inflammatory responses and inflammatory mediators. When our bodies are invaded by fungi, lipids will move to the location of the lesions, mobilizing the immune system to resist it [17,18].

All metabolites in this study were potential targets of CMK as fungicides. N-acetylsphingosine has been identified in this study, playing a key role as an intermediate in sphingolipid metabolism. The phosphorylation product of sphingosine, known as sphinganine-1-phosphate (S1P), is closely associated with various inflammatory diseases and cytokines [19,20,21]. S1P and its receptors actively participate in regulating the body’s inflammatory responses [22,23,24]. In the ether lipid metabolism pathway, dihydroxyacetone phosphate Ester is converted into 1-alkyl-glycerone-3-phosphate using alkyl dihydroxyacetone phosphate synthase, thereby exerting immunomodulatory, anti-inflammatory, and antifungal. 5-androstenediol, a steroid hormone, can stimulate the immune system and bone marrow leukocytes to resist the pathogenic microorganisms [25].

The tricarboxylic acid (TCA) cycle and the metabolism of glyoxylate and dicarboxylic acid play a crucial role in energy metabolism. The TCA cycle, also known as the citric acid cycle, is a fundamental metabolic pathway in aerobic organisms. Glyoxylate and dicarboxylic acid metabolism are essential steps allowing fatty acids to enter the TCA cycle. We have discovered that the CMK extract can upregulate the TCA cycle, as well as interfere with glyoxalic acid and dicarboxylic acid metabolism. The regulation of the TCA cycle process is primarily achieved by changing the levels of citric acid and cis-aconitic acid. Citrate is a TCA cycle metabolite that accumulates in activated immune cells. Mitochondrial citrate is converted into isocitrate via cis-aconitate by the enzyme aconitase of the TCA cycle. These two metabolites can increase the anti-inflammatory response of immune cells [26]. Palsson-McDermott EM et al. demonstrated that TCA cycle metabolism in anti-inflammatory immune cells is significantly upregulated in inflammatory tissues, such as CD8^+^T cells and M2 macrophages [27].

## 4. Materials

The reagents, equipments, and animals are shown in the Supplementary Materials, specifically Tables S1–S3.

## 5. Methodology

### 5.1. Extraction of Carex meyeriana Kunth

The CMK powder (90 g) was refluxed three times with 70% ethanol, for 1 h each time. The filtrate was then combined, concentrated, and diluted with deionized water. The minimal inhibitory concentration (MIC) was determined using the broth microdilution method. The inhibition rate of CMK against *C. albicans* was calculated as follows:

Inhibition rate (Y) = [1 − (A_experimental group_ − A_blank_)/(A_control_ − A_blank_)] × 100%.

### 5.2. Identification of Ingredients in Carex meyeriana Kunth Extracts

The total chemical components of the CMK ethanol extracts and the chemical constituents in the *C. albicans* lysates (the lysis method for *C. albicans* can be found in Supplementary Materials 1.3, Tables S4 and S5) were analyzed using ultra-performance liquid chromatography–quadrupole tandem time-of-flight mass spectrometry (UPLC-Q-TOF-MS^E^). The chromatographic and mass spectrometry conditions are also detailed in Supplementary Materials 1.4 and 1.5. Data were collected using MassLynx V4.1 software, matched with a database derived from the UNIFI data platform (a chemical database developed by the Waters^TM^ instrument workstation, USA). Compounds with an error of less than 5 ppm were selected for standard comparison, secondary ion mass spectrometry fragment information verification, and documentation, and were then combined with the mass spectrometry fragmentation patterns to infer the chemical composition and source. Three traditional Chinese medicine standards of *Tricin*, *Luteolin*, and *Myricetin* were used for verification of flavonoids. (The linearity of the standards was very good, *R*^2^ > 0.999, Supplementary Materials Figures S6–S8.)

### 5.3. Vaginitis Rats Were Infected with C. albicans

Rats with *C. albicans* vaginitis were used based on the standard procedure method described in refs. [28,29,30].

(1) Pretreatment: After 1 week of adaption, 0.5 mL/100 g estradiol benzoate for veterinary purposes was injected subcutaneously into each rat daily for 2 days. After 6 days, the drug-induced rat estrus model was successfully established, and then the rats were injected every 2 days until the end of the experiment.
(2) Intravaginal inoculation: One day after successfully establishing a drug-induced estrus model in rats, 1.5 × 108 CFU/mL of C. albicans suspension was spread over the vagina mucosa at a dose of 200 μL for each rat. The control group was given an equal amount of sterile water. The procedure was repeated daily for 4 days. The evaluation criteria for a successful infection of C. albicans included visible congestion, edema, and increased secretion of the vulva and vaginal mucosa to the naked eye, as well as fungi (+) in the vaginal secretions.
(3) Drug treatment: All rats were randomly divided into five groups: model group (vaginitis due to C. albicans), control group (2 mL of 0.9% sodium chloride solution), fluconazole (FCZ) group (the C. albicans vaginitis rats were treated with 13.5 mg/kg fluconazole), a high-dose group (the *C. albicans* vaginitis rats were treated with 8 g/kg of CMK extract), and a low-dose group (the *C. albicans* vaginitis rats were treated with 2 g/kg of CMK extract), with eight rats in each group. Medication was applied to the vaginal area. Drug treatment continued for 14 consecutive days.

### 5.4. Numbers of C. albicans Colonies

WE used 0.9% sodium chloride solution to repeatedly rinse the vaginas of rats, and a total of 1 mL of vaginal lavage solution was collected from the day before and 2, 5, 8, 11, and 14 days after medication. The collected samples were centrifuged at 6000 r/min for 10 min; then, the supernatant was diluted. Subsequently, 100 μL of the diluted solution was inoculated into LB culture medium and incubated at 37 °C for 24 h. The resulting colonies (CFU/mL) were then counted.

### 5.5. Inflammatory Mediators in the Vaginal Tissue

The levels of IL-4, IL-6, IL-10, IL-1β, and TNF-α from vaginal tissue were measured using double antibody sandwich ELISA after continuous administration of the treatment for 14 days.

### 5.6. Vaginal Histopathological Staining

HE and PAS staining were performed to investigate the effect of CMK on rats’ vaginal tissues after its continuous administration for 14 days.

### 5.7. Metabolomics Analysis

The samples (the collection method for which is shown in the Supplementary Materials 1.6 and 1.7) included the vaginal pathological tissues of rats and *C. albicans* (10^3^ CFU/mL). UPLC-Q-TOF-MS was utilized for detection (the conditions can be found in the Supplementary Materials 1.8, Tables S6 and S7). The raw data were analyzed using progenesis QI software v3.0. Subsequently, EZinfo2.0 software was employed for multivariate statistical analysis. Principal component analysis (PCA) and orthogonal partial least-squares differentiation analysis (OPLS-DA) were used to analyze the changes in the metabolic profiles of each group and to screen for potential biomarkers. Compounds with VIP > 1 and *p* < 0.05 were identified as differential metabolites. Then, the information about the metabolites was matched with the information in the HMDB database (http://www.hmdb.ca/, accessed on 4 February 2024). Finally, identification and enrichment analysis of the metabolic pathways was conducted using the KEGG (http://www.kegg.com, accessed on 4 February 2024) and MetaboAnalyst 5.0 (http://www.metaboanalyst.ca, accessed on 4 February 2024) databases.

### 5.8. Statistical Analysis

All data were analyzed using GraphPad Prism 7, and significant differences between groups were analyzed using *t*-tests and LSD methods.

## 6. Conclusions

Our experimental results demonstrate that CMK not only exhibits strong anti-*C. albicans* properties but also exhibits an anti-inflammatory effect by regulating multiple metabolic targets and metabolic pathways, showing promising clinical application prospects. In conclusion, as a widely distributed plant in northeast China, CMK has the potential to effectively treat vaginitis caused by *C. albicans*.

## Figures and Tables

**Table 1:** The chemical components derived from *C. albicans* lysates.

NO.	Components	t_R_/min	Formula	Calculated Mass (*m*/*z*)	Found Mass (*m*/*z*)	Error (ppm)	Adducts	Fragment (*m*/*z*)
1	Neotigogenin acetate	7.90	C_12_H_46_O_4_	459.3504	458.3396	3.5	+H	217.1353
2	Luteolin	10.99	C_15_H_10_O_6_	287.0571	286.0477	2.1	+H	117.1326
3	Aschantin	18.94	C_22_H_24_O_7_	401.1601	400.1522	0.6	+H	383.1541, 401.1440
4	Echinothiophene	18.99	C_23_H_26_O_10_S	495.1292	494.1247	−2.8	+H	475.1083, 493.1315
5	Trifolin	6.98	C_21_H_20_O_11_	447.0937	448.1006	0.4	−H	284.0319
6	Orientin	7.02	C_21_H_20_O_11_	447.0945	448.1006	1.3	−H	285.0397, 297.0378, 299.0555, 327.0535, 357.2607
7	Rutin	7.47	C_27_H_30_O_16_	609.1482	610.1534	2.1	−H	300.1355
8	Tracheloside	8.29	C_27_H_34_O_12_	549.1974	550.2050	−0.4	−H	550.2015
9	β-Hydroxyisovalerylshikonin	9.64	C_21_H_24_O_7_	433.1512	388.1522	0.8	+HCOO	225.0732, 252.1370, 338.2455
10	Tiliroside	11.12	C_30_H_26_O_13_	593.1322	594.1373	2.2	−H	255.0274, 284.0324, 285.0409
11	Plantamajoside	11.06	C_29_H_36_O_16_	685.1979	640.2003	−0.7	+HCOO	477.1230
12	Sinensetin	12.54	C_20_H_20_O_7_	371.1139	372.1201	0.3	−H	328.0594
13	Isodemethylwedelolactone	14.25	C_15_H_8_O_7_	299.0206	300.0270	0.9	−H	271.0264, 227.0337, 199.0376, 201.0170, 283.0131, 187.0002, 183.0425
14	Tricin	14.93	C_17_H_14_O_7_	329.0673	330.0740	0.6	−H	329.0673, 314.0434, 271.0248, 227.0340, 185.0205, 161.0198
15	Isosilybin	15.17	C_26_H_24_O_10_	495.1297	496.1370	0.0	−H	477.3274
16	Wedelolactone	17.92	C_16_H_10_O_7_	313.036	314.0427	0.7	−H	299.0204
17	9,16-Dioxyhydroxy-10,12,14-triene-18 carbonic acid	18.47	C_18_H_30_O_4_	309.2059	310.2144	−1.2	−H	211.0763
18	6-Gingerol	20.16	C_17_H_26_O_4_	293.1785	294.1831	2.6	−H	293.1780
19	Methyl lucidenate P	23.92	C_30_H_44_O_8_	531.3007	532.3036	4.4	−H	531.3003

**Table 2:** Metabolites identified in vaginal tissue.

Mode	NO.	t_R_ (min)	Formula	Measured Mass (*m*/*z*)	Proposed Identity	CON/MOD	HD/MOD	LD/MOD
ESI+	1	0.67	C_6_H_6_O_4_	165.0110	Sumiki’s acid	↓ ****	↓ ***	↓ *
	2	0.78	C_8_H_7_NO_3_	166.0426	4-Pyridoxolactone	↓ ***	↓ **	↓
	3	3.52	C_19_H_39_NO_3_	330.3102	Palmitoyl Serinol	↓ **	↓ *	↓ **
	4	3.55	C_21_H_30_O_2_	337.2057	7-Dehydropregnenolone	↓ *	↓ ***	↓ *
	5	3.58	C_21_H_41_NO_3_	356.3294	Pristanoylglycine	↑	↑ **	↑
	6	3.60	C_19_H_30_O_2_	313.2026	5-Androstenediol	↑ **	↑	↓
	7	3.72	C_4_H_7_O_7_P	199.0095	Dihydroxyacetone Phosphate Acyl Ester	↓ ****	↓	↑
	8	4.46	C_19_H_26_O_2_	286.4085	Androst-4-ene-3,17-dione	↑	↑ *	↑
	9	4.80	C_20_H_32_O_6_	368.2258	6,15-Diketo,13,14-dihydro-PGF1a	↑	↓ ****	↓
	10	6.79	C_26_H_43_NO_6_	466.3181	Glycocholic acid	↑ **	↓ ***	↓ *
	11	7.36	C_19_H_32_O	299.2216	5a-Androstan-3b-ol	↑	↑ *	↑
	12	7.39	C_20_H_34_O_5_	354.2460	Prostaglandin F2a	↑ ****	↑ ****	↑ ***
	13	14.24	C_36_H_71_NO_3_	566.5704	N-Acylsphingosine	↑ ***	↑	↓
ESI−	14	0.65	C_3_H_6_O_4_	142.9751	Glyceric acid	↑	↑ *	↑
	15	0.72	C_6_H_8_O_7_	191.0194	Citric acid	↑ **	↑ *	↑
	16	1.30	C_2_H_2_O_3_	72.9931	Glyoxylic acid	↓	↓ *	↓
	17	2.82	C_8_H_16_N_2_O_3_	187.1090	N6-Acetyl-L-lysine	↓	↓ *	↓
	18	2.88	C_22_H_32_O_3_	343.2351	19(20)-EpDPE	↓ *	↓ **	↓
	19	2.99	C_22_H_30_O_5_	373.2152	11β,20-Dihydroxy-3-oxopregn-4-en-21-oic acid	↓	↓ ***	↓
	20	3.23	C_22_H_32_O_2_	327.2387	Docosahexaenoic acid	↑ **	↑	↓
	21	3.41	C_6_H_6_O_6_	173.0022	cis-Aconitic acid	↓	↓ *	↓ *
	22	3.67	C_25_H_34_O_8_	461.2159	6-Dehydrotestosterone glucuronide	↑	↑ ****	↓
	23	3.72	C_19_H_38_O_2_	343.2713	Nonadecanoic acid	↓ **	↓ ***	↓
	24	4.13	C_21_H_34_O_4_	349.2259	5a-Tetrahydrocorticosterone	↓ *	↓	↓ *
	25	4.49	C_24_H_38_O_5_	405.2675	7-Ketodeoxycholic acid	↓ ***	↓ ***	↓
	26	6.08	C_18_H_35_NO_3_	312.2468	Palmitoylglycine	↓	↓ **	↓

Note: Compared to the control group, * denotes a statistically significant increase or decrease in metabolites. The greater the number of *, the larger the statistical difference observed. ↓: down; ↑: up.

**Table 3:** Metabolites of *C. albicans* identified.

Mode	NO.	t_R_ (min)	Formula	Measured Mass (*m*/*z*)	Proposed Identity	CMK/CON
ESI+	1	0.43	C_21_H_28_O_5_	361.1924	Aldosterone	↓ **
	2	0.56	C_14_H_23_N_6_O_3_S	378.1431	S-Adenosylmethioninamine	↑ *
	3	0.97	C_3_H_7_NO_2_S	121.1586	L-Cysteine	↓ *
	4	1.16	C_13_H_22_N_4_O_8_S_2_	449.0710	Cysteineglutathione disulfide	↓ ****
	5	1.68	C_19_H_28_O_3_	327.2012	16-Oxoandrostenediol	↓ **
	6	2.60	C_24_H_40_O_7_S	472.2553	Chenodeoxycholic acid 3-sulfate	↑ ***
	7	4.01	C_19_H_30_O_3_	329.2136	5-Androstenetriol	↓ ****
	8	5.53	C_22_H_45_NO	340.3487	Docosanamide	↓ *
	9	7.35	H_2_O_3_S_2_	114.1445	Thiosulfate	↑ **
	10	8.28	C_10_H_14_N_5_O_10_PS	450.0111	Adenosine phosphosulfate	↑ *
	11	8.90	C_7_H_14_N_2_O_4_S	223.0655	Allocystathionine	↓ ***
	12	9.69	C_7_H_15_N_2_O_8_P	286.0493	Glycineamide ribotide	↓ ****
	13	10.13	C_26_H_45_NO_6_S	499.3147	Taurochenodesoxycholic acid	↓ ***
	14	12.20	C_17_H_33_NO_4_	316.2619	Decanoylcarnitine	↓ ***
	15	12.40	C_8_H_17_NOS_2_	230.0593	Dihydrolipoamide	↓ *
ESI−	16	0.95	C_14_H_11_Cl_2_NO_3_	356.0077	4′-Hydroxydiclofenac	↑ *
	17	6.52	C_26_H_32_O_14_	613.1773	Phloretin xylosyl-galactoside	↑ ****
	18	11.33	C_10_H_12_N_4_O_6_	329.0666	Xanthosine	↑ ****
	19	12.54	C_24_H_38_O_4_	390.2764	9′-Carboxy-alpha-chromanol	↓ *
	20	12.65	C_11_H_14_O_4_	209.0820	3,4-Dihydroxyphenylvaleric acid	↓ ****
	21	12.94	C_10_H_13_N_4_O_8_P	348.2062	Inosinic acid	↑ **
	22	13.48	C_15_H_22_O_2_	279.1617	Valerenic acid	↓ ****
	23	13.99	C_7_H_12_N_2_O_6_	265.0702	L-β-aspartyl-L-serine	↑ **

Note: Compared to the control group, * denotes a statistically significant increase or decrease in metabolites. The greater the number of *, the larger the statistical difference observed. ↓: down; ↑: up.

## Data Availability

The authors declare that the data supporting the findings of this study are available within the paper and its Addition file. Should any raw data files be needed in another format they are available from the corresponding author upon reasonable request.

## Supplementary Materials

The following supporting information can be downloaded at: https://www.mdpi.com/article/10.3390/ijms25137288/s1.

## References

[1] Sobajo O.A., Okiki P.A., Ade-Ojo I.P., Adelegan O., Adarabioyo M.I. (2022). *Candida albicans* virulence genes SAP1 and SAP6 associated with vaginitis among pregnant women attending a Nigerian tertiary hospital. New Microbes New Infect..

[2] Yang Y., Su L., Liu Y., Zhuang X., Wang R., Zhang Y. (2019). Study on the efficacy of rehabilitation new fluid against Candida vaginitis. Mod. Chin. Med. China.

[3] Ji J., Jiang Y., Han S. (2015). In vitro antagonism of *C. albicans*. World Sci. Technol.-Mod. Tradit. Chin. Med..

[4] De Freitas A.L.D., Kaplum V., Rossi D.C.P., da Silva L.B.R., Melhem M.D.S.C., Taborda C.P., de Mello J.C.P., Nakamura C.V., Ishida K. (2018). Proanthocyanidin polymeric tannins from *Stryphnodendron adstringens* are effective against *Candida* spp. isolates and for vaginal candidiasis treatment. J. Ethnopharmacol..

[5] Li Z., Tan X., Wang G., Chen C., Ma Y., Astulla A., Liu C., Yang J., He X. (2023). To explore the scientific connotation of traditional Chinese medicine based on “drug-ingredients-target”. Chin. Herb. Med..

[6] Qi J., Liu X., Zhang Y., Zhu G., Tang S., Yu X., Su Y., Chen S., Liang D., Chen G. (2023). Adsorption of chloramphenicol from water using *Carex meyeriana* Kunth-derived hierarchical porous carbon with open channel arrays. Environ. Sci. Pollut. Res. Int..

[7] Su T. (2021). Study on the Antibacterial Active Substances and the Mechanism of Action.

[8] Wang H. (2020). Analysis of the Chemical Composition of Flavonoids and Its Antioxidant and Antibacterial Activity.

[9] Sousa Y.V., Santiago M.G., de Souza B.M., Keller K.M., Oliveira C.S., Mendoza L., Vilela R.V., Goulart G.A. (2024). Itraconazole in human medicine and veterinary practice. J. Mycol. Med..

[10] Fang J., Huang B., Ding Z. (2021). Efficacy of antifungal drugs in the treatment of oral candidiasis: A Bayesian network meta-analysis. J. Prosthet. Dent..

[11] Sobel J. (2023). Role of Antifungal Susceptibility tests in the treatment of vulvovaginal candidiasis. Curr. Infect. Dis. Rep..

[12] Akdağ D., Pullukçu H., Yamazhan T., Metin D.Y., Sipahi O.R., Ener B., Tasbakan M.I. (2019). Fluconazole-Resistant *Candida albicans* Vaginitis with Cross-Resistance to Azoles: A Case Report. Open Forum Infect. Dis..

[13] Akdağ D., Pullukçu H., Yamazhan T., Metin D.Y., Sipahi O.R., Ener B., Tasbakan M.I. (2021). Anidulafungin treatment for fluconazole-resistant *Candida albicans* vaginitis with cross-resistance to azoles: A case report. J. Obs. Gynaecol..

[14] Zida A., Bamba S., Yacouba A., Ouedraogo-Traore R., Guiguemdé R.T. (2017). Anti-*Candida albicans* natural products, sources of new antifungal drugs: A review. J. Mycol. Med..

[15] Gao M., Wang H., Zhu L. (2016). Quercetin assists fluconazole to inhibit biofilm for mations of fluconazole-resistant *Candida albicans* in in vitro and in vivo antifungal managements of vulvovaginal candidiasis. Cell Physiol. Biochem..

[16] Cheng X., Cheng Y., Zhang N., Zhao S., Cui H., Zhou H. (2020). Purification of flavonoids from *Carex meyeriana* Kunth based on AHP and RSM: Composition analysis, antioxidant, and antimicrobial activity. Ind. Crops Prod..

[17] Bosch M., Sánchez-Álvarez M., Fajardo A., Kapetanovic R., Steiner B., Dutra F., Moreira L., López J.A., Campo R., Marí M. (2020). Mammalian lipid droplets are innate immune hubs integrating cell metabolism and host defense. Science.

[18] Bosch M., Pol A. (2022). Eukaryotic lipid droplets: Metabolic hubs, and immune first responders. Trends Endocrinol. Metab..

[19] Tsai H.C., Han M.H. (2016). Sphingosine-1-phosphate (S1P) and S1P signaling pathway: Therapeutic targets in autoimmunity and inflammation. Drugs.

[20] Kunkel G.T., Maceyka M., Milstien S., Spiegel S. (2013). Targeting the sphingosine-1-phosphate ate axis in cancer, inflammation and beyond. Nat. Rev. Drug Discov..

[21] Hannun Y.A., Obeid L.M. (2018). Sphingolipids and their metabolism in physiology and disease. Nat. Rev. Mol. Cell Biol..

[22] Hou L., Yang L., Chang N., Zhao X., Zhou X., Dong C., Liu F., Yang L., Li L. (2020). Macrophage sphingosine 1-phosphate receptor 2 blockade attenuates liver inflammation and fibrogenesis triggered by NLRP3 inflammasome. Front. Immunol..

[23] Park E.S., Chol S., Shin B., Yu J., Yu J., Hwang J.M., Yun H., Chung Y.H., Choi J.S., Choi Y. (2015). Tumor necrosis factor (TNF) receptor-associated factor (TRAF)-interacting protein (TRIP) negatively regulates the TRAF2 ubiquitin-dependent pathway by suppressing the TRAF2-sphingosine 1-phosphate (S1P) interaction. J. Biol. Chem..

[24] Kitano M., Hla T., Sekiguchi M., Kawahito Y., Yoshimura R., Miyazawa K., Iwasaki T., Sano H. (2006). Sphingosine 1-phosphate/sphingosine 1-phosphate receptor 1 signaling in rheumatoid synovium: Regulation of synovial proliferation and inflammatory gene expression. Arthritis Rheum..

[25] Whitnall M.H., Inal C.E., Jackson W.E., Miner V.L., Villa V., Seed T.M. (2001). In vivo Radioprotection by 5-androstenediol: Stimulation of the innate immune system. Radiat. Res..

[26] Williams N.C., O’Neill L.A.J. (2018). A Role for the Krebs cycle intermediate citrate in metabolic reprogramming in innate immunity and inflammation. Front. Immunol..

[27] Pålsson-McDermott E.M., O’Neill L.A.J. (2020). Targeting immunometabolism as an anti-inflammatory strategy. Cell Res..

[28] Jia L. (2021). Effect of Crude Extract of Rostro-Tail Pipa Nail on Autophagy in Rat Aerobic Vaginitis.

[29] Fatahi Dehpahni M., Chehri K., Azadbakht M. (2021). Effect of Silver Nanoparticles and L-Carnitine Supplement on Mixed Vaginitis Caused by *Candida albicans*/*Staphylococcus aureus* in Mouse Models: An Experimental Study. Curr. Microbiol..

[30] Zhang R. (2023). Study on the Anti-Inflammatory Effect and Mechanism of Glucomannan Gel Treatment for Mixed Infectious Vaginitis and Cervicitis in Rats.

