# Supplementary material for: Carex meyeriana Kunth Extract Is a Novel Natural Drug against Candida albicans
Source: Int J Mol Sci. 2024 Jul 2;25(13):7288. doi: 10.3390/ijms25137288 (PMC11242224; doi:10.3390/ijms25137288)
Supplement: Supplementary file 1 [file ijms-25-07288-s001.zip › ijms-3032210-supplementary.pdf]

1 Supplementary Methods

1.1 Equipments

Table S1. Main equipments

equipments	Manufacturer
Ultra clean workbench	Shanghai Boxun Industrial Co., Ltd.,China
Milli-Q Gradient A10 ultrapure water system	Millipore, Bedford, Massachusetts, USA
SYNAPT G2-S ultra-high performance liquid chromatography	Waters, Milford, Massachusetts, USA
Q-TOF SYNAPT G2 HDMS high-resolution mass spectrometer	Waters, Milford, Massachusetts,USA

1.2 Drugs and reagents

Table S2. Main drugs and reagents

Drugs and reagents	Manufacturer
Candida albicans standard strain	Beona Innovation Alliance Biotech Inc. BNCC299343,China.
Carex meyeriana Kunth	Jilin Liaoyuan Donglin straw weaving Co., Ltd, China
Fluconazole	Pfizer Inc., New York,USA(NO.FG4909)
Tricin, Luteolin, Myricetin standard material	Shanghai Fusion Medicine Technology Co., Ltd,China
Luria-Bertani culture medium	Qingdao Haibo Biotechnology, China
Candida Chromatic Agar Reference Medium	Qingdao Haibo Biotechnology, China
ELISA kits	Youersheng Biotechnology, China
PAS and HE staining kits (NO.BA4080A, BA-4097, BA-4022)	Zhuhai Besso Biotechnology, China

Table S3. Animal

species	number	Weeks of age	Weight (g)	Manufacturer	License number
SPF SD female rat	30	8	180～220	Liaoning Changsheng Biotechnology Co., LTD.China	SCXK (Liao) 2020-0001.

1.3 The drug treatment method

2ml of *C. albicans* suspension was added to 100ml of LB medium for 2-3 hours. Following this, the concentration of the *C. albicans* was adjusted to 10^3 CFU/mL, and CMK alcohol extract was added for 6, 12, and 24 hours. Control group was added with PBS buffer solution. There were 3 replicates per group. The *C. albicans* solution was transferred into sterile centrifuge tubes and centrifuged at 4℃, 12000 r/min for 5 minutes. The supernatant was discarded, and the precipitates were washed and resuspended with PBS. The same procedure was repeated 3 times.

The precipitate was mixed with lysis reagents and the *C. albicans* cells were ultrasonically broken at a 70% power setting (on for 3 seconds/off for 5 seconds) for a total time of 2 minutes. After ultrasonic treatment, centrifuge at 4℃ and 12000 r/min for 2 min. The supernatant was retained and the precipitation was treated with 70% anhydrous ethanol. Another round of ultrasonic crushing is performed under the same conditions. Finally, all supernatants are collected. The same procedure was repeated 3 times. Finally, the collected supernatant(*C. albicans* lysates) was dried by nitrogen.

1.4 Chromatographic conditions:

(1) The conditions for determining the total chemical composition from CMK:C18 chromatography column (1.7 μm, 2.1 × 50 mm, Waters™, USA) are as follows:

- Column temperature: 30℃
- Sample room temperature: 4℃
- Injection volume: 5 μL
- Flow rate: 0.3 mL/min
- Mobile phase: A (acetonitrile) - B (0.1% formic acid water)

The gadient elution procedure was as follows:

Table S4. The conditions for gradient elution

Time(min)	Mobile phase A volume fraction%	Mobile phase B volume fraction%
0	5	95
1	10	90
3	50	50
6	60	40
7	61	39
9	70	30
12	90	10
14	100	0

(2) The conditions for determining chemical components from *C. albicans* lysates:ChromCore™120 C18 chromatography column (3.0×100 mm ,3 μm, NanoChrome™ ,UK)are as follows:

-Column temperature :30℃,

-Mobile phase : acetonitrile(A)-0.1 %formic acid water(B),

-Flow rate of :0.3 mL/min,

-Injection volume of :10 μL.

The gadient elution procedure was as follows:

Table S5. The conditions for gradient elution

Time(min)	Mobile phase A volume fraction%	Mobile phase B volume fraction%
0	5	95
3	15	85
7	30	70
11	35	65
15	40	60
20	60	40
22	78	22
24	95	5
25	100	0
26	100	0
27	5	95
29	5	95

1.5 Mass spectrum conditions:

(1) The conditions for determining the total chemical composition from CMK are as follows: Ion fragments information is collected under positive and negative modes using ESI ion sourse. In MS scanning mode, the mass scanning range is 50-1000 m/z with a scanning time of 0.5 s. The capillary voltage is set at 2.5 kV in the negative ion mode and 2.8 kV in the positive ion mode, while the source temperature is maintained at 100℃ and the desolvent temperature at 350℃. The flow rate of conical hole gas (N_2) is set to 35.0 L/h, and the flow rate of desolvent gas (N_2) to 500.0 L/h. Additionally, the gas flow rate of the atomizer is set at 6.3 Bar;the cone voltage at 20 V, with real-time correction performed using leucine enkephalin.

(2) The conditions of chemical components in *C. albicans* lysate were as follows: Ion fragments information is collected under positive and negative modes using ESI ion sourse. In MS scanning mode, the scanning time was 0.2s, the mass scanning range is 50~1200Da. The ion source temperature is 120℃, and the desolvent gas temperature is 300℃. The flow rate of conical hole gas (N_2) is 50.0 L/h, and the flow rate of dissolved gas (N_2) is 600 L/h. The capillary voltage is set to 3.0 kV and the cone hole voltage is set to 40 kV. A calibrated solution of 0.2 ng/L leucine enkephalin was used for analysis.

1.6 Methods for collecting vaginal tissue

① After the rats were anesthetized, more than 10mL of blood was extracted from the abdominal aorta until respiration and cardiac activity in the rats ceased. After the needle was extracted, the rat's vaginal tissue was dissected.

② The bilateral ovaries of the rats and their connected Y-shaped uterus were located in the abdominal cavity. The lower end of the uterus contained a lumen-shaped vagina, approximately 1.5cm in length. When the vagina was separated downward, It was necessary to use scissors to cut the bilateral pubis of the rat, fully exposed the vagina area.

③ The reproductive tract of the rat was completely dissected, including the vaginal opening. Carefully using scissors to separate the connective tissue attached around the vagina from the bladder and ureter located in front of the vagina.

④ Make a longitudinal incision upward from the vaginal opening. A double cervical opening can be observed approximately 1.5cm from the vaginal opening. The vaginal tissue was fully opened, revealing a rectangular shape measuring approximately 1.0cm*1.5cm.

⑤The specimen is then vertically divided into three equal parts. One part is immersed in a 10% neutral formaldehyde solution for the preparation of pathological sections, while the other two parts are frozen at -80℃. Of the two frozen sections, one is designated for ELISA testing and the other for metabolomics analysis.

1.7 Methods for collecting *C. albicans*

① 2 mL *C. albicans* solution was inoculated into 100 mL of LB liquid medium for 2-3 hours, resulting in a concentration of 10^3 CFU/mL. Subsequently, the alcohol extract of CMK was added and left to incubate overnight. A control group without the alcohol extract of CMK was also prepared. Each group was arranged with three parallel samples.

② The *C. albicans* solution was centrifuged at 12000 r/min for 5 minutes at 4℃, the supernatant discarded, and the sediment retained.

③ The sediment was washed with PBS, then centrifuged again at 12000 r/min for 5 minutes at 4℃.The procedure was repeated three times.

④ The sediment was mixed with lysate reagents, swirled for 1 minute, and then subjected to low temperature ultrasonication (3 seconds on, pause for 5 seconds, repeated for a total of 2 minutes at a power level of 70%). Subsequently, it was centrifuged at 12000 r/min for 2 minutes at 4℃; the supernatant was discarded and the sediment retained.

⑤ A solution containing 70% ethanol was added and rotated, followed by another round of centrifugation at 4℃ (12000 r/min, 2 minutes) to collect the supernatant.

⑥ The entire process was repeated twice, followed by the mixing of all the collected supernatant and subsequent nitrogen drying. Prior to UPLC-Q-TOF-MS analysis, an appropriate amount of methanol was added to redissolve the dried substance. Subsequently, the solution was centrifuged at 4℃ (12000 r/min) for 10 minutes, and the resulting supernatant was used for analysis.

1.8 Metabolomics analysis

1.8.1 Vaginal tissue samples were analyzed using UPLC-Q-TOF-MS

(1) Chromatographic conditions:

-Waters C18 column(1.7 μm, 2.1×50 mm)

-Column temperature :30℃,

-Mobile phase : acetonitrile(A)-0.1 %formic acid water(B),

-Flow rate of :0.3 mL/min,

-Injection volume of :5 μL.

The gadient elution procedure was as follows:

Table S6. The conditions for gradient elution

Time(min)	Mobile phase A volume fraction%	Mobile phase B volume fraction%
0	5	95
1	10	90
3	50	50
6	60	40
7	61	39
9	70	30
12	90	10
14	100	0

(2) Mass spectrum conditions:

Electrospray ion source was used to collect ion debris information under positive and negative scanning modes. In MS scanning mode, the mass scanning range was 50-1000 m/z, the scanning time was 0.5 s, the capillary voltage was 2.5 kV in negative ion mode, 2.8 kV in positive ion mode, the source temperature was 100℃, and the desolvent temperature was 350℃. The cone-hole gas flow rate was 35.0 L /h, the desolvent gas flow rate was 500.0 L /h, the atomizer gas flow rate was 6.3 Bar, and the cone-hole voltage was 20 V. Real-time correction was adopted by leucine enkephin.

1.8.2 *Candida albicans* samples were analyzed using UPLC-Q-TOF-MS

(1) Chromatographic conditions:

-Waters C18 column(1.7 μm, 2.1×50 mm)

-Column temperature :30℃,

-Mobile phase : acetonitrile(A)-0.1 %formic acid water(B),

-Flow rate of :0.3 mL/min,

-Injection volume of :5 μL.

The gadient elution procedure was as follows:

Table S7. The conditions for gradient elution

Time(min)	Mobile phase A volume fraction%	Mobile phase B volume fraction%
0	5	95
3	20	80
6	25	75
9	27	73
11	30	70
13	45	55
15	100	0

(2) Mass spectrum conditions:

Electrospray ion source was used to collect ion debris information under positive and negative scanning modes. In MS scanning mode, the mass scanning range was 50-1000 m/z, the scanning time was 0.5 s, the capillary voltage was 2.5 kV in negative ion mode, 2.8 kV in positive ion mode, the source temperature was 100℃, and the desolvent temperature was 350℃. The cone-hole gas flow rate was 35.0 L /h, the desolvent gas flow rate was 500.0 L /h, the atomizer gas flow rate was 6.3 Bar, and the cone-hole voltage was 20 V. Real-time correction was adopted by leucine enkephin.

Table S8. Chemical composition of CMK in positive ion mode

NO.	Component	t_R /min	Formula	Calculated mass (m/z)	Found mass (m/z)	Error (ppm)	Adducts	Fragment (m/z)
**Flavonoids**								
5	Sophoraflavonoloside	4.50	$C_{27}H_{30}O_{16}$	610.1534	611.1597	-0.9	+H	449.1091、593.1486
7	Rutin	5.28	$C_{27}H_{30}O_{16}$	610.1534	611.1611	0.4	+H	609.1462
10	APIGENIN-6-ARABINOSIDE-8-GLUCOSIDE	5.49	$C_{26}H_{28}O_{14}$	564.1480	565.1546	-0.5	+H	457.1101、529.1328、547.1434、565.1544、
11	Leucocyanidin	5.67	$C_{15}H_{14}O_7$	306.0740	329.0644	1.3	+Na	163.0308、271.0983
14	Cianidanol	6.57	$C_{15}H_{14}O_6$	290.0790	313.0703	2.0	+Na	121.0168、123.0341
18	Sophoraflavanone B	7.40	$C_{20}H_{20}O_5$	340.1311	341.1365	-1.8	+H	137.0500、187.0703、271.1000、301.1243、323.0904
19	Hydroxygenkwanin	7.64	$C_{16}H_{12}O_6$	300.0634	301.0697	-1.0	+H	153.0100、167.0276、227.1723、229.0464、241.0473、258.0512、269.0423、285.0376、286.0435
21	Genistein-1	8.35	$C_{15}H_{10}O_5$	270.0528	271.0592	-0.9	+H	153.0105
22	Diosmetin	8.49	$C_{16}H_{12}O_6$	300.0634	301.0704	-0.3	+H	257.0434、286.0463、299.1376
23	Baicalin	9.04	$C_{21}H_{18}O_{11}$	446.0849	447.0916	-0.6	+H	177.0486
27	Procyanidin C-1	10.14	$C_{45}H_{38}O_{18}$	866.2058	889.2001	5.1	+Na	287.0535、453.1217
28	Luteolin	11.52	$C_{15}H_{10}O_6$	286.0477	287.0547	-0.3	+H	117.0220、145.0557
30	Axillarin	11.93	$C_{17}H_{14}O_8$	346.0689	347.0764	0.3	+H	153.0103
32	Isosilybin	13.49	$C_{26}H_{24}O_{10}$	496.1370	497.1438	-0.4	+H	283.0976、301.1074、463.0924
46	Icaritin	22.34	$C_{21}H_{20}O_6$	368.1260	369.1340	0.7	+H	297.0396、313.0716
**Organic acids**								
6	Adoxosidic acid	5.26	$C_{16}H_{24}O_{10}$	376.1370	377.1452	0.9	+H	151.0309
8	Gingerone	5.29	$C_{11}H_{14}O_3$	194.0943	217.0834	-0.1	+Na	137.0503、163.0675、167.0638
12	Rubianic acid	6.15	$C_{25}H_{26}O_{13}$	534.1373	535.1447	0.0	+H	517.1342
16	Sebacic acid	7.06	$C_{10}H_{18}O_4$	202.1205	225.1086	-1.1	+Na	139.0436、201.0489
31	Gypsogenic acid	12.92	$C_{30}H_{46}O_5$	486.3345	487.3409	-0.9	+H	105.0567、119.0738、131.0740
34	Sanleng acid	14.35	$C_{18}H_{34}O_5$	330.2406	353.2303	0.4	+Na	128.0517、145.0922、295.2264
37	Salvianolic acid A	16.85	$C_{26}H_{22}O_{10}$	494.1213	495.1278	-0.8	+H	179.0282、313.0704
39	13-Hydroxy-9,11-hexadecadienoic acid	18.06	$C_{16}H_{28}O_3$	268.2038	291.1945	1.4	+Na	291.1952
43	Palmitoleic acid	20.55	$C_{16}H_{30}O_2$	254.2246	277.2147	0.9	+Na	153.0605、167.0700
47	Myristic acid	22.56	$C_{14}H_{28}O_2$	228.2089	251.1979	-0.2	+Na	145.0556、155.0777、197.1270
**Phenylpropanoids**								
4	Schininallylol	4.19	$C_{20}H_{24}O_5$	344.16240	367.1504	-1.2	+Na	193.0761、207.0639、367.1496
9	Isolariciresinol	5.39	$C_{20}H_{24}O_6$	360.1573	383.1483	1.8	+Na	145.0548、163.0675、193.0444、325.1358、345.1373
25	Podophyllotoxin	9.26	$C_{22}H_{22}O_8$	414.1315	415.1374	-1.4	+H	313.0731
29	Ningposide A	11.65	$C_{18}H_{22}O_9$	382.1264	405.1188	3.2	+Na	131.0742、175.0312
33	Weelolactone	13.76	$C_{16}H_{10}O_7$	314.0427	315.0496	-0.3	+H	231.0605、259.0552、285.0386
38	Cnidimol D	17.37	$C_{15}H_{16}O_6$	292.0947	315.0864	2.5	+Na	276.0581、315.0948
40	Aschantin	18.57	$C_{22}H_{24}O_7$	400.1522	401.1590	-0.5	+H	383.1498、401.1580
44	Ningposide C	20.69	$C_{17}H_{20}O_8$	352.1158	375.1074	2.4	+Na	164.0392、169.0945、187.0340、229.0471、315.0467
50	Futoquinol	23.60	$C_{21}H_{22}O_5$	354.1467	355.1548	0.8	+H	175.1059、189.0860
**Terpenoids**								
13	Clerodendrin	6.36	$C_{27}H_{26}O_{17}$	622.1170	623.1238	-0.5	+H	623.1233
26	(-)-Istanbulin A	9.62	$C_{15}H_{20}O_4$	264.1362	287.1274	2.0	+Na	153.0584、207.0615
35	13,17-Epoxy alisol A	14.36	$C_{30}H_{50}O_6$	506.3607	507.3683	0.3	+H	277.2149
48	Methyl lucidenate Q	22.67	$C_{28}H_{42}O_6$	474.2981	497.2881	0.8	+Na	285.0385、497.2877

49	3-O-(2′E,4′Z-Decadienoyl) ingenol	23.50	$C_{30}H_{42}O_6$	498.2981	499.3042	-1.2	+H	521.3428
**Others**								
1	Heterodendrin	1.98	$C_{11}H_{19}NO_6$	261.1212	262.1272	-1.3	+H	136.0537
2	Cordycepin	2.07	$C_{10}H1_3N_5O_3$	251.1018	274.0906	-0.5	+Na	136.0537、234.0727
3	Adenosine	2.74	$C_{10}H_{13}N_5O_4$	267.0968	268.1027	-1.3	+H	136.0527、268.1028
15	β-Hydroxyisovalerylshikonin	7.05	$C_{21}H_{24}O_7$	388.1522	389.1585	-0.9	+H	225.1084、241.0474、269.0417
17	Neotigogenin acetate	7.09	$C_{29}H_{46}O_4$	458.3396	459.3440	-2.9	+H	121.0546、217.0816
20	Anisodamine	7.92	$C_{17}H_{23}NO_4$	305.1627	306.1691	-0.9	+H	149.0513
24	2′-Acetylacteoside	9.09	$C_{31}H_{38}O_{16}$	666.2160	689.2059	0.7	+Na	137.0502
36	Cerevisterol	16.23	$C_{28}H_{46}O_3$	430.3447	453.3362	2.3	+Na	217.0438、453.3358
41	Echinothiophene	18.63	$C_{23}H_{26}O_{10}S$	494.1247	495.1272	-4.7	+H	476.1090、493.1392
42	Cnidimol B	20.12	$C_{15}H_{16}O_6$	292.0947	315.0865	2.5	+Na	217.0470
45	Aurantiamide acetate	21.97	$C_{27}H_{28}N_2O_4$	444.2049	445.2139	1.8	+H, +Na	105.0207、117.0588、194.1102
51	(E,E)-9-Oxooctadeca-10,12-dienoic acid	23.86	$C_{18}H_{30}O_3$	294.2195	317.2111	2.4	+Na	121.0171、293.2107

Table S9. Chemical composition analysis of CMK in negative ion mode

NO.	Components	t_R /min	Formula	Calculated mass (m/z)	Found mass (m/z)	Error (ppm)	Adducts	Fragment (m/z)
**Flavonoids**								
4	Procyanidin B6	5.21	$C_{30}H_{26}O_{12}$	578.1424	577.1369	1.7	-H	289.0709、407.0777、577.1374
5	Eriocitrin	5.82	$C_{27}H_{32}O_{15}$	596.1741	595.1662	-0.7	-H	163.0385、269.0497、447.0944、451.0588
6	Apigenin6-C-arabinoside-8-glucoside	6.69	$C_{26}H_{28}O_{14}$	564.1479	563.1406	0.0	-H	161.0234、297.0783、353.0681、383.0783、443.1000、503.1197、545.1319
8	Trifolin	6.87	$C_{21}H_{20}O_{11}$	448.1006	447.0934	0.1	-H	285.0417、269.0466
9	Orientin	6.89	$C_{21}H_{20}O_{11}$	448.1006	447.0934	0.1	-H	285.0417、297.0376、299.0559、300.0302、327.0535、339.0519、357.0460、429.0832
10	L-Epicatechin	6.92	$C_{15}H_{14}O_6$	290.0790	335.0779	0.6	+HCOO	109.0264、151.0021、271.0265
12	Rutin	7.04	$C_{27}H_{30}O_{16}$	610.1534	609.1471	1	-H	609.1462、300.1027
18	Clerodendrin	7.48	$C_{27}H_{26}O_{17}$	622.1170	621.1111	1.4	-H	351.0586
30	aceosidin	8.88	$C_{17}H_{14}O_7$	330.0740	329.0677	1.1	-H	299.0681、314.0445
31	5-Methylkaempferol	9.06	$C_{16}H1_2O_6$	300.0634	299.0571	1.0	-H	285.0402
33	Luteolin-7-O-glucuronide	9.34	$C_{21}H_{18}O_{12}$	462.0798	461.0749	2.3	-H	285.0412
35	Tiliroside	10.79	$C_{30}H_{26}O_{13}$	594.1373	639.1356	0.1	+HCOO	255.0078、284.0342、285.0078
37	Isorhamnetin	11.78	$C_{16}H_{12}O_7$	316.0583	315.0526	1.6	-H	151.0023、243.0315、300.0278
38	Luteolin	11.82	$C_{15}H_{10}O_6$	286.0477	285.0418	1.3	-H	107.0108、133.0273、151.0020、217.0510、257.0473、267.0306、285.0417
39	Sinensetin	12.14	$C_{20}H_{20}O_7$	372.1209	371.1147	1.1	-H	297.0415、329.2357、339.0545

41	Nobiletin	12.63	$C_{21}H_{22}O_8$	402.1315	401.1250	0.8	-H	109.0252、327.2194、343.1135
43	Tricin	14.23	$C_{17}H_{14}O_7$	330.0740	329.0680	1.3	-H	329.0681、314.0447、271.0264、227.0356、185.0241、161.0234
44	Naringenin	14.53	$C_{15}H_{12}O_5$	272.0685	271.0621	0.9	-H	119.0472、151.0379
45	Isosilybin	14.74	$C_{26}H_{24}O_{10}$	496.1370	495.1300	0.3	-H	477.1180
50	Myricetin	16.71	$C_{15}H_{10}O_8$	318.0326	317.0311	-0.7	-H	271.0360、151.0016、137.0352
52	Kuwanon L	16.91	$C_{35}H_{30}O_{11}$	626.1788	671.1771	0.1	+HCOO	309.2082、269.0486
54	Kushenol V	17.56	$C_{21}H_{22}O_7$	386.1366	385.1303	1.0	-H	193.0499、177.0185
61	5-Hydro-7,8,2'-trimethoxyflavanone	20.80	$C_{18}H_{16}O_6$	328.0947	373.0938	0.9	+HCOO	271.0232、285.0422
**Organic acids**								
1	Quinic acid	1.79	$C_7H_{12}O_6$	192.0634	191.0559	-0.2	-H	87.0046、127.0371、191.0559
2	Citric acid	2.03	$C_6H_8O_7$	192.0270	191.0199	0.1	-H	173.0088、128.0331、111.0059、87.0047
3	1-O-Caffeoyquinic acid	4.93	$C_{16}H_{18}O_9$	354.0951	353.0890	1.2	-H	353.0872
13	Shikimic acid	7.08	$C_7H_{10}O_5$	174.0528	173.0448	-0.7	-H	93.0305、111.0416、137.0218、155.0333
15	Isochlorogenic acid C	7.29	$C_{25}H_{24}O_{12}$	516.1268	561.1281	3.2	+HCOO, -H	179.0348、191.0563、335.0555、353.0683、515.1277
16	Rubianic acid	7.32	$C_{25}H_{26}O_{13}$	534.1373	533.1300	-0.1	-H	413.0888、473.1112
17	Sebacic acid	7.35	$C_{10}H_{18}O_4$	202.1205	247.1194	0.7	+HCOO	139.0021
20	Pimelic acid	7.54	$C_7H_{12}O_4$	160.0736	159.0652	-1.0	-H	115.0734
23	E-p-Coumatic acid	8.25	$C_9H_8O_3$	164.0473	163.0390	-1.1	-H	93.0307、119.0469
26	Isochlorogenic acid B	8.42	$C_{25}H_{24}O_{12}$	516.1268	515.1197	0.2	-H	175.0984
28	Isochlorogenic acid A	8.76	$C_{25}H_{24}O_{12}$	516.1268	515.1200	0.5	-H	137.0221
46	Sanleng acid	14.78	$C_{18}H_{34}O_5$	330.2406	329.2344	1.0	-H	329.2345、229.1450、211.1340、183.1388、171.1018
48	Pentadecanoic acid	15.90	$C_{15}H_{30}O_2$	242.2246	287.2236	0.8	+HCOO	269.0480
56	Salvianolic acid A	17.70	$C_{26}H_{22}O_{10}$	494.1213	493.1140	0.0	-H	133.0271、177.0185、295.2212
57	9,16-Dioxyhydroxy-10,12,14-triene-18 carbonic acid	17.97	$C_{18}H_{30}O_4$	310.2144	309.2082	1.1	-H	309.2083、171.1019、211.1334
58	Tianshic acid	18.75	$C_{18}H_{34}O_5$	330.2406	329.2341	0.7	-H	185.1175、211.1335
64	Terminalic acid	22.72	$C_{20}H_{28}O_3$	316.2038	315.1981	1.5	-H	317.2120
**Phenylpropanoids**								
11	Ningposide C	6.99	$C_{17}H_{20}O_8$	352.1158	397.1136	-0.4	+HCOO	315.1248、357.1355
14	Cistanoside F	7.19	$C_{21}H_{28}O_{13}$	488.1530	487.1492	3.5	-H	163.0381、427.1099
21	Gomisin G	7.66	$C_{30}H_{32}O_9$	536.2046	581.2004	-2.5	+HCOO	325.0747、372.1191、505.1271
22	Tracheloside	8.17	$C_{27}H_{34}O_{12}$	550.2050	595.2025	-0.7	+HCOO	387.1148
25	Urolignoside	8.32	$C_{26}H_{34}O_{11}$	522.2101	521.2015	-1.4	-H	331.0685
40	Schisantherin A	12.41	$C_{30}H_{32}O_9$	536.2046	581.2015	-1.3	+HCOO	373.1284
**Terpenoids**								
24	1-Deoxyeucommiol	8.31	$C_9H_{16}O_3$	172.1099	217.1088	0.7	+HCOO	171.1029、217.1094
27	Blumenol C glucoside	8.63	$C_{19}H_{32}O_7$	372.2148	417.2139	0.9	+HCOO	193.0129、209.0433、371.0810、373.1322
29	Albiflorin R1	8.77	$C_{23}H_{28}O_{11}$	480.1632	525.1647	3.4	+HCOO	134.0348、283.1045
34	Eucommiol	9.47	$C_9H_{16}O_4$	188.1049	187.0974	-0.2	-H	187.0988、125.0950
47	Mudanpioside D	15.27	$C_{24}H_{30}O_{12}$	510.1737	509.1696	3.1	-H	134.0348、509.1685
49	Androsta-4-ene-3,17-dione	16.54	$C_{19}H_{26}O_2$	286.1933	331.1928	1.3	+HCOO	331.1924
62	Preleoheterin	22.21	$C_{20}H_{30}O_4$	334.2144	333.2082	1.1	-H	317.2126
65	Methyl lucidenate P	23.65	$C_{30}H_{44}O_8$	532.3036	531.2968	0.5	-H	531.2964
**Others**								
7	Amurenlactone A	6.86	$C_{17}H_{20}O_9$	368.1107	413.1099	1.0	+HCOO	135.0427、191.0558
19	Sibirioside A	7.53	$C_{21}H_{28}O_{12}$	472.1581	471.1547	3.9	-H	190.0633、471.1539

32	β-Hydroxyisovalerylshikonin	9.33	$C_{21}H_{24}O_7$	388.1522	387.1442	-0.7	+HCOO, -H	225.0732、251.1302
36	Plantamajoside	10.82	$C_{29}H_{36}O_{16}$	640.2003	685.1998	1.3	+HCOO	297.0405、477.1152、639.1365
42	Isodemethylwedelolactone	14.21	$C_{15}H_8O_7$	300.0270	299.0215	1.8	-H	271.0264、227.0356、199.0402、201.0196、283.0277、187.0028、183.0448
51	Glycyrrhizic acid	16.82	$C_{42}H_{62}O_{16}$	822.4038	821.3974	0.9	-H	113.0217、193.0479、351.0590、821.3977
53	Citronellyl formate	16.96	$C_{11}H_{20}O_2$	184.1463	229.1450	0.5	+HCOO	229.1457
55	Wedelolactone	17.60	$C_{16}H_{10}O_7$	314.0427	313.0370	1.7	-H	299.0222、315.0489
59	6-Gingerol	19.71	$C_{17}H_{26}O_4$	294.1831	293.1765	0.6	-H	177.0187
60	Falcarindiol	20.41	$C_{17}H_{24}O_2$	260.1776	305.1769	1.1	+HCOO	161.0243、149.0222
63	Dibutyl sebacate	22.28	$C_{18}H_{34}O_4$	314.2457	313.2392	0.7	-H	313.2393、201.1129

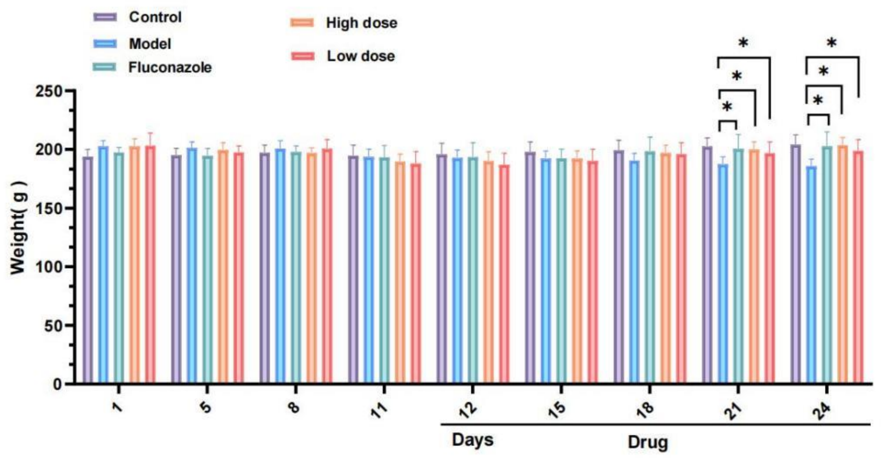

Figure S1. Weight of rats. After 21 days of drug treatment, the rats' weight gradually recovered. Compare with Model group,*P<0.05.

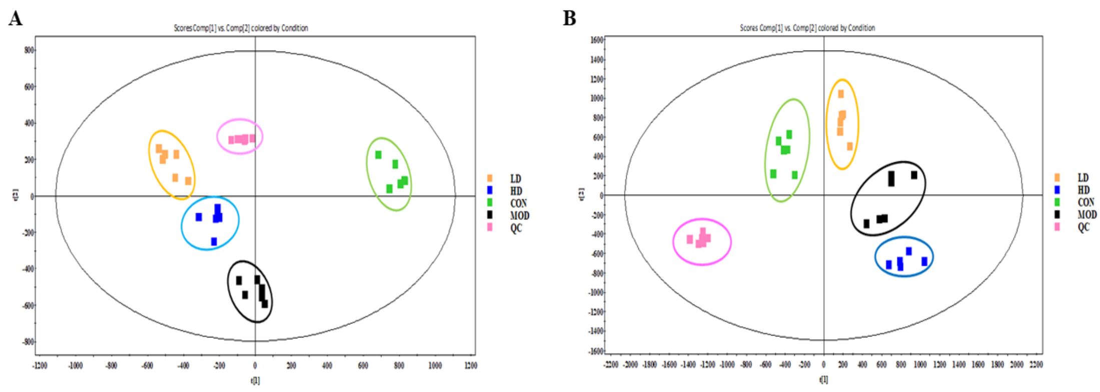

Figure S2. Metabonomics of vaginal mucosa:PCA scores. (A) positive ion modes; (B)negative ion modes. The samples within each group are clustere, but there was dispersion of inter-group samples by principal component analysis.

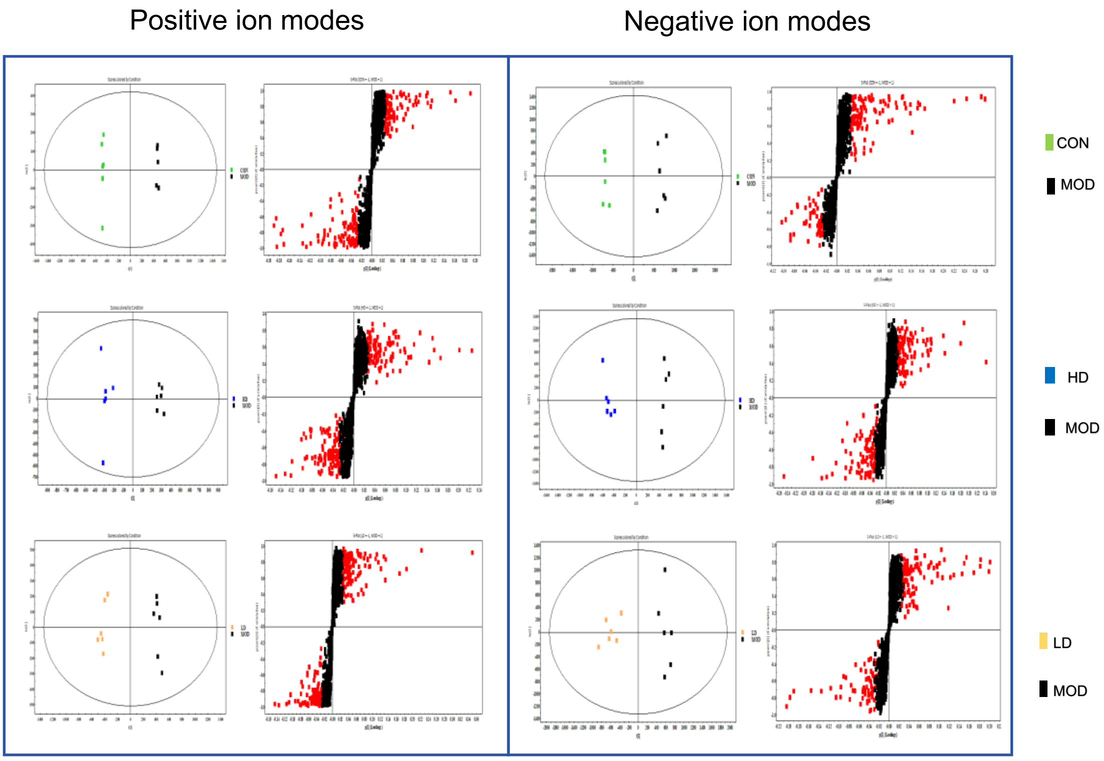

Figure S3. Metabonomics of vaginal mucosa:S-Plot base on OPLS-DA analysis. in positive and negative ion modes. The diagrams of the model group and other groups in positive and negative ion mode demonstrate significant separation. R2Y and Q2 are utilized to assess the suitability and predictive capability of the model. In positive ion mode of HPLC-ESI-MSn, R2Y=99% and Q2=97% in the CON and MOD groups, while in negative ion mode, R2Y=98% and Q2=94%, indicating substantial differences in endogenous metabolites between the CON group and the MOD group. The model exhibits good predictive ability and fitting degree. VIP>1.0 and P<0.05 are identified as potential biomarker candidates.

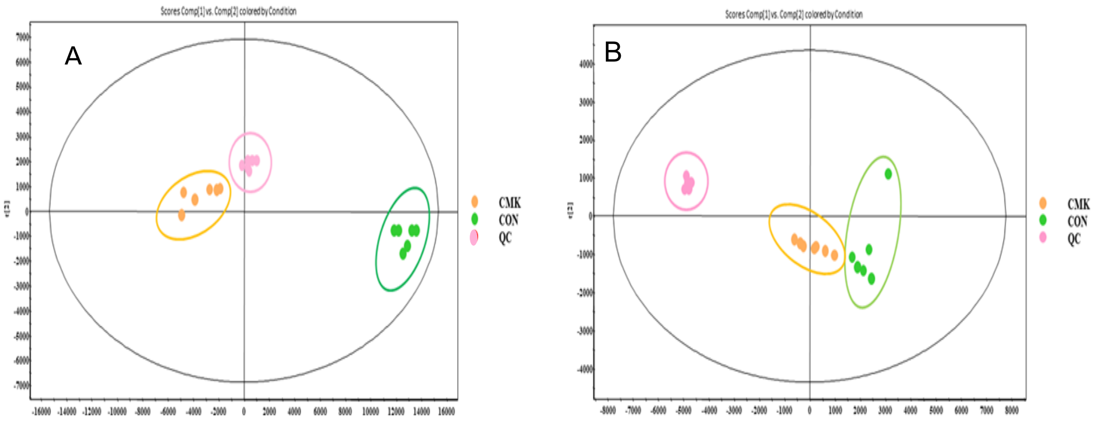

Figure S4. Metabonomics of *Candida albicans:*PCA scores.(A) positiveand ion modes; (B)negative ion modes. The blank control group (CON) and the CMK group show significant separation, while the CMK group show a trend closer to the CON group. Samples within each group are cluster, while samples between groups were significantly separate, indicating that different metabolic profiles of *Candida albicans*.

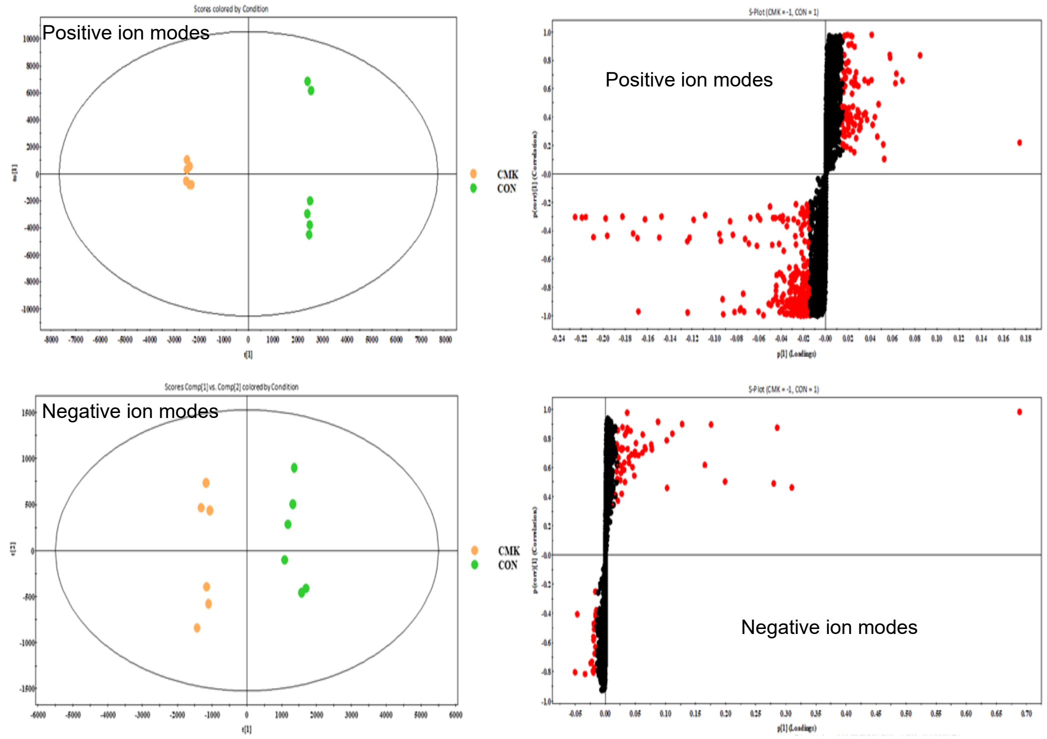

Figure S5. Metabonomics of *Candida albicans:*S-Plot based on OPLS-DA analysis. The diagrams of the model group and other groups in positive and negative ion mode demonstrate significant separation. R2Y and Q2 are utilized to assess the suitability and predictive capability of the model. The R2Y=99% and Q2=97% in the CON and CMK groups in positive ion mode of HPLC-ESI-MSn, while the R2Y=94% and Q2=90% in negative ion mode, indicating substantial differences in endogenous metabolites between the CON group and the CMK group. The model exhibits good predictive ability and fitting degree. VIP>1.0 and P<0.05 serve as potential biomarker candidates.

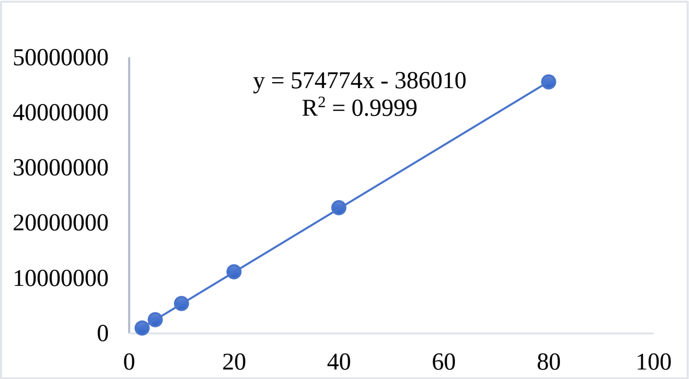

Figure S6. Standard curve of *luteolin*

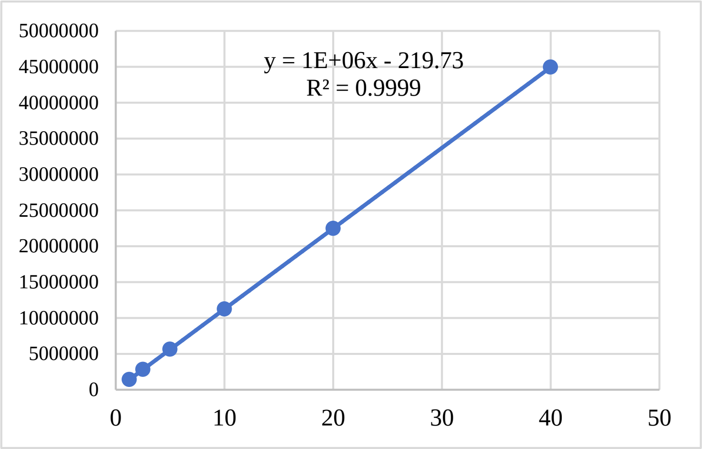

Figure S7. Standard curve of *Myricetin*

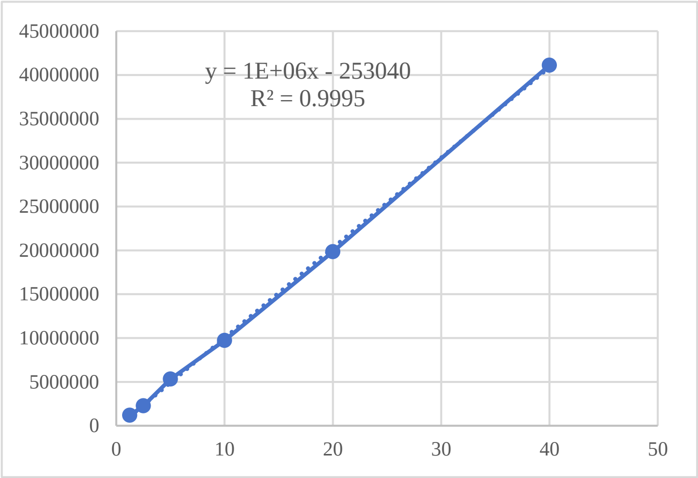

Figure S8. Standard curve of *Tricin*
